# Anti-Icing Property of Superhydrophobic Nanostructured Brass via Deposition of Silica Nanoparticles and Nanolaser Treatment

**DOI:** 10.3390/nano13071139

**Published:** 2023-03-23

**Authors:** Saqib Hussain, Tanyakorn Muangnapoh, Bhawat Traipattanakul, Milin Lekmuenwai

**Affiliations:** 1 School of Manufacturing Systems and Mechanical Engineering, Sirindhorn International Institute of Technology, Thammasat University, Pathum Thani 12120, Thailand; 2 National Nanotechnology Center (NANOTEC), National Science and Technology Development Agency (NSTDA), Pathum Thani 12120, Thailand

**Keywords:** superhydrophobic, brass, silica nanoparticles, nanolaser, anti-icing, droplet, icing time

## Abstract

Ice accumulation on brass surfaces can lead to heat transfer inefficiency, equipment degradation, and potential accidents. To address this issue, superhydrophobic surface technology is utilized. This work aims to develop superhydrophobic nanostructured brass surfaces using the combination of nanolaser ablation and the deposition of silica nanoparticles to achieve the anti-icing property. Four distinct types of brass surfaces namely, the bare surface (BS), the lasered surface (LS), the coated surface (CS), and the coated-lasered surface (CLS) were prepared. The anti-icing performances of the fabricated samples including the effects of the surface structure, the droplet size, and the surface temperature were investigated and evaluated. The results showed that the delayed icing time increased with the increases in the apparent contact angle, the droplet size, and the surface temperature. When the apparent contact angle increased, the contact area between the droplet and the cooling substrate reduced, leading to the longer delayed icing time. With the deposition of silica nanoparticles and nanolaser treatment, CLS achieved the greatest apparent contact angle of 164.5°, resulting in the longest delayed icing time under all experimental conditions. The longest delayed icing time on CLS recorded in this study was 2584 s, which was 575%, 356%, and 27% greater than those on BS, LS, and CS, respectively. The study also revealed that the surface structure played a more crucial role in achieving the anti-icing property when compared to the surface temperature or the droplet size. The shortest delayed icing time on CLS at the lowest surface temperature and at the smallest droplet size was longer than those on BS and LS at all conditions. The results were also discussed in relation to a heat transfer model. The findings of this research can serve as an avenue for advancing knowledge on heat transfer enhancement and energy efficiency.

## 1. Introduction

Ice buildup is a major issue in refrigeration and air conditioning systems, the aviation industry, electrical systems, and other industrial applications. Frost and ice aggregation on external surfaces of equipment in low-temperature conditions can cause lower equipment performance efficiency, economic loss, and serious security problems. For example, ice development on aircraft surfaces induced by supercooled droplets in the atmosphere alters airflow over the wings and the tail, causing aerodynamic stalls which can potentially result in a sudden loss of control of the aircraft [1]. Furthermore, significant ice adhesion to metal and alloy surfaces can damage equipment and reduce efficiency [2]. 

Among several surface coating solutions [3,4,5,6,7,8,9,10,11,12], superhydrophobic surface coating technology has been proven to be one of the effective methods to address ice buildup issues [6,7,8,9,10,11,12]. Inspired by nature [13,14,15,16], a surface that is superhydrophobic must meet three criteria: (i) the apparent contact angle is greater than 150°, (ii) the contact angle hysteresis is less than 10°, and (iii) the Cassie wetting state is highly stable [17]. Unlike Wenzel’s state, the Cassie Baxter model is used to describe a droplet resting on the tip of the composite air-solid surface. Since the droplet does not touch the surface of the substrate, the air pockets between the surface structures below the droplet increase, leading to a decrease in the contact area between the water droplet and the solid substrate, which also results in an increase in the contact angle and a low droplet adhesion [18,19]. With the small solid-liquid contact area, heat transfer between the substrate and the droplet reduces, and the freezing process of liquid droplets on the surface is delayed [20,21]. Given the definition of icephobicity, surfaces with good anti-icing properties can prevent ice buildup either from dropwise condensation droplets or incoming droplets, and have low adhesion between the ice and the surface [17]. Therefore, superhydrophobic technology has been widely utilized in anti-icing applications. However, it must be noted that not all superhydrophobic surfaces can possess excellent anti-icing properties due to small voids at the liquid–solid interface [22]. Apart from the anti-icing purposes, superhydrophobic surfaces can also be applied in a number of applications including self-cleaning [23,24], anti-fogging [25], heat transfer enhancement [26,27], corrosion resistance [28] and oil-water separation [29]. 

Superhydrophobic surfaces can be fabricated with various methods such as spin coating, solution immersion, chemical etching, the layer-by-layer method, and chemical vapor deposition [30,31,32,33,34]. Li et al. [6] fabricated an anti-icing superhydrophobic surface on magnesium alloy using a single-step hydrothermal technique, and tested the anti-icing property at −15 °C. The coated surface showed an apparent contact angle of 156.7°. The water droplet stayed unfrozen for 150 s on the bare surface, whereas the droplet on the superhydrophobic surface solidified into ice after 600 s. Zuo et al. [7] fabricated an anti-icing superhydrophobic aluminum surface via chemical etching and hot-water treatment. The test temperature was set at −6 °C. Droplets on a bare surface completely turned into ice after 187 s, whereas droplets on a fabricated surface were fully frozen after 207 min. The coated surface gave the apparent contact angle of 164.8°. Bao et al. [8] fabricated a superhydrophobic composite coating based on hydrangea-like ZnO@Cus on aluminum alloy. The sample was tested at −15 °C and the delayed icing time was 13 min. The apparent contact angle of the coated surface was found to be 163.5°. In addition to surface coating, different micro-/nanostructures resulted in different anti-icing performances. Liu et al. [9] fabricated different superhydrophobic surface structures on 7075 aluminum alloy samples with a fiber laser and the stearic acid solution. The study showed that the superhydrophobic structure with the greatest apparent contact angle resulted in the longest delayed icing time. 

In addition to the metal materials stated above, brass, a non-ferrous metal consisting of copper alloy and zinc, is used in several industrial applications. Icing issues on brass surfaces are inevitable. Thus, developing a superhydrophobic surface on brass can address these problems. Jian et al. [10] fabricated a nanostructured superhydrophobic brass surface modified with low surface energy materials. At the surface temperature of −15 °C, the micro/nanoscaled hierarchical structured superhydrophobic sample with the apparent angle of 157.50° had a delayed icing time of 3657 s. Qing et al. [11] prepared a superhydrophobic surface using sandpaper as a template, and a hydrothermal reaction was used to create modified titanium dioxide nanoparticles. An apparent contact angle of 154° was reported. The fabricated surface was glued on a brass substrate. After covering the surface with snow for 12 h, the sample was tilted, and the snow slid off the surface. Wang et al. [12] created a layered double hydroxide (LDH) coating on a brass substrate. The apparent contact angle was found to be 160° for the coated surface. The findings showed that the droplet on the coated surface took 45 min to be fully frozen at the temperature of −10 °C.

Although a few past studies utilized superhydrophobic brass for anti-icing purposes, the combined fabrication methods of silica nanoparticle deposition and nanolaser treatment on a brass substrate, and its anti-icing performance have never been reported before. Additionally, there is a lack of understanding of several parameters including the surface structure, the surface temperature, and the droplet size which affect the anti-icing performance of superhydrophobic nanostructure on brass substrates. Thus, in this study, superhydrophobic nanostructures were created on brass substrates using nanolaser processing, deposition of silica nanoparticles, and a combination of nanolaser processing and deposition of silica nanoparticles. Furthermore, the effects of surface structures, surface temperatures, and droplet sizes on the anti-icing capabilities of bare brass, lasered brass, coated brass, and coated-lasered brass samples were investigated and compared. The results under different experimental settings were also explained in terms of heat transfer mechanisms. This is the first study in which nanolaser processing and silica nanoparticle deposition were carried out to fabricate superhydrophobic structures on brass substrates for anti-icing applications. The findings of the study can shed light on how to solve ice accumulation issues on brass in various conditions. Surface fabrication and surface characterization methods are described in Section 2, and the experimental setup is discussed in Section 3. In addition, the experimental results of the study and the analysis are shown and explained in Section 4. Then, the conclusion is drawn in Section 5.

## 2. Materials 

### 2.1. Surface Fabrication 

Four brass sheets (50 mm × 25 mm × 1 mm) were used as substrates in this study. Four different types of brass samples which were the bare surface (BS), the lasered surface (LS), the coated surface (CS), and the coated-lasered surface (CLS) were fabricated and experimentally tested. The brass sheets were polished with sandpaper with #500, #1000, and #1500 grit sizes, and then cleaned with isopropyl alcohol and kept in an ultrasonic bath for 10 min at which point brass sheets with BS were obtained and three of them were later modified into the other surfaces. To prepare LS, a polished brass sample was treated with Marking Laser 20 W with a frequency of 30 kHz, scanning speed of 300 mm/s, spot diameter of 0.05 mm, a laser power range of 90%, and three loops. With the nanolaser treatment, groove patterns were created on the brass surface. To fabricate CS, a coating solution consisting of silica nanopowder, tetraethyl orthosilicate (TEOS), isopropyl alcohol (IPA), ammonium hydroxide, and tridecafluorooctyl triethoxy silane (FAS) was prepared. Firstly, TEOS was mixed with IPA, and the mixture was stirred for 30 min. Ammonium hydroxide solution was then added to the solution. After that, the silica nanopowder was added to the mixture and the mixture was stirred again. Later, FAS was added to the mixture to generate a functional group and enhance the bonding towards the substrate, and the mixture was stirred for 6 h. The TEOS-FAS superhydrophobic solution was sprayed on a polished brass substrate. It should be noted that although the prepared solution can be applied to a substrate with several coating techniques, the results from the study conducted by Adamopoulos et al. [35] showed that spraying gave the best non-wetting property. To prepare CLS, a polished brass sample was lasered and then sprayed with the TEOS-FAS superhydrophobic solution.

### 2.2. Surface Characterization

A contact angle device (KRUSS, 230 V Mobile Surface Analyzer) with a water droplet volume of 10 µL at the ambient temperature was used to measure the apparent contact angle of each sample. The apparent contact angle was measured at three different positions on each substrate and their average values were recorded. Additionally, to ensure superhydrophobicity, a contact angle instrument (Dataphysics, OCA40) was used to measure the advancing and receding contact angles of CS and CLS. The measurement method from [36] was adopted, and measurements were performed three times on each substrate. The contact angle hysteresis was calculated from the difference between the advancing angle and the receding contact angle. The average contact angle hysteresis of CS and CLS were recorded. In addition, in order to observe the structure of the surface after laser processing, a 3D microscope was used to visualize the structure of LS and CLS. The morphologies of the prepared surfaces of all samples were examined with scanning electron microscopy (SEM). Moreover, the chemical compositions of the samples were analyzed with energy-dispersive spectroscopy (EDS).

## 3. Experimental Setup

**Figure 1:**
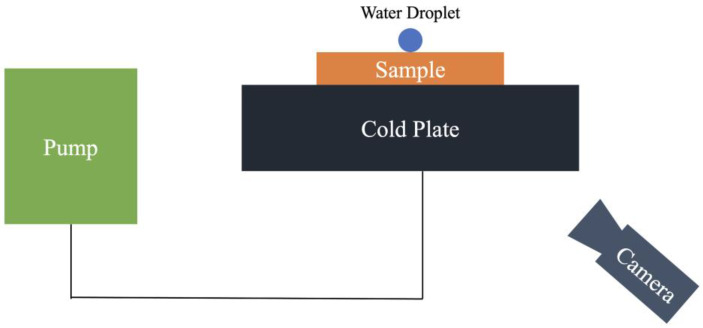
Schematic Diagram of the Experimental Setup.

An apparatus consisting of a cold plate, a pump, and a camera was used to conduct the experiment as shown in Figure 1. The cold plate was used to control the temperature of the samples during the anti-icing test, and its temperature was monitored with a thermocouple device. The water droplet size was controlled with micropipettes. Prior to the anti-icing experiment, the samples were kept in an oven at 30 °C to maintain the initial temperature. The cold plate temperature was set, a sample was moved to the cold plate, and the experiment was carried out and recorded with the camera. The delayed icing times of water droplets were measured at three different surface temperatures at −5 °C, −10 °C, and −15 °C for each sample. At each temperature, droplet sizes of 10 µL, 20 µL, and 30 µL were used. A nitrogen gun was used to blow away condensates on the samples after the tests. After each test, the sample was transferred to the oven before the next set of the experiment. The delayed icing time is defined as the time between the placing of a water droplet on a sample and the point at which the droplet turned into ice completely. The experiment was performed at an ambient condition with a temperature of 24 °C, and a relative humidity of approximately 55%. The uncertainties of the experiment included the cold plate power (±5%), the thermocouple device (±2%), the ambient temperature and humidity (±4%), and the droplet volume from the micropipettes (±0.1%). From the error propagation of multiplication [37], the total uncertainty of this experiment accounted for 6.7%. 

## 4. Results and Discussion

### 4.1. Surface Morphology and Wetting Properties

**Figure 2:**
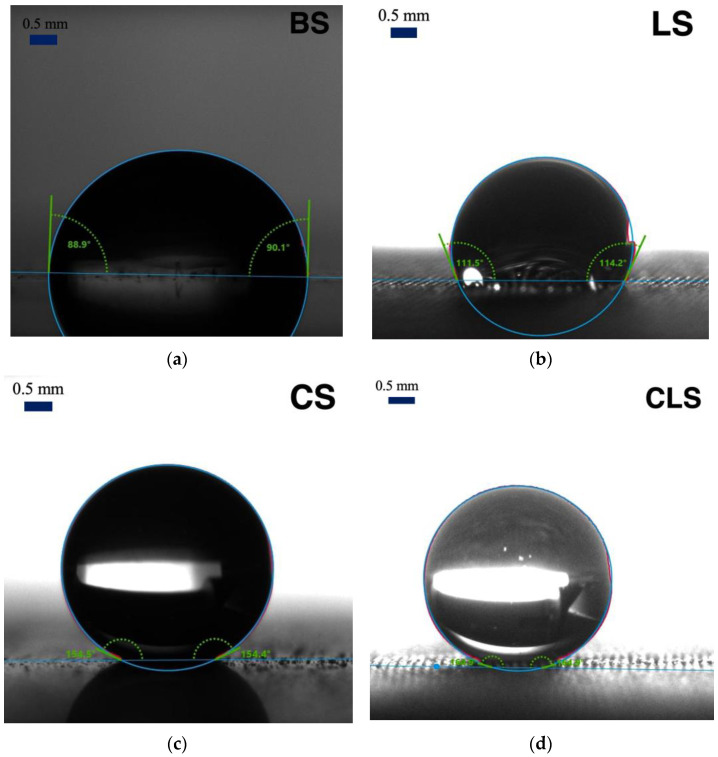
Contact angle measurements of brass substrate with (**a**) BS, (**b**) LS, (**c**) CS, and (**d**) CLS.

**Figure 3:**
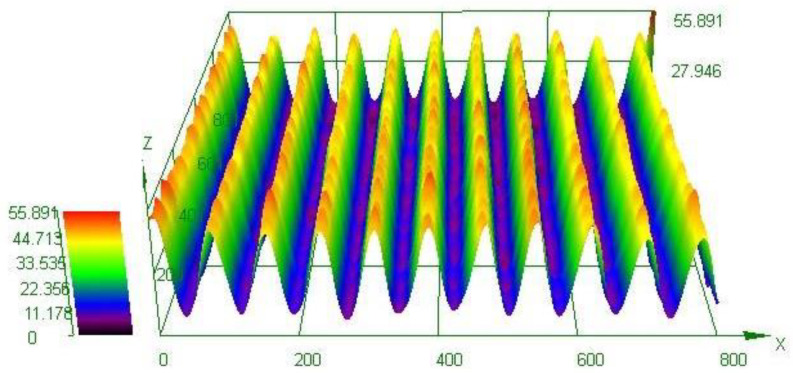
3D microscopic image of the brass substrate with nanolaser treatment.

**Figure 4:**
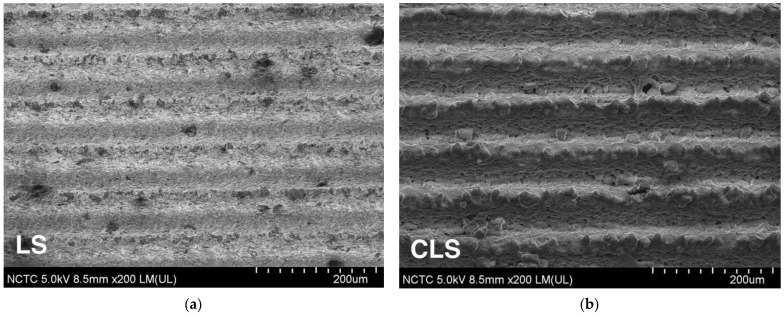

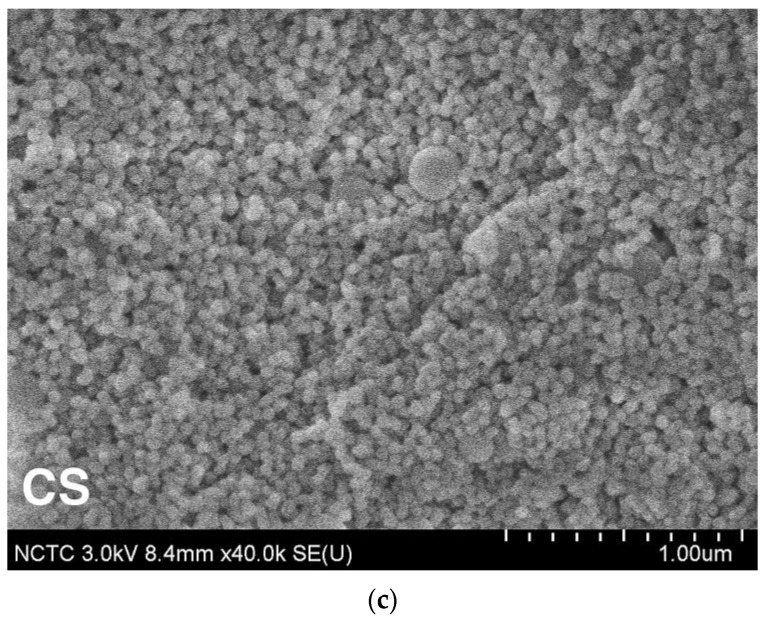
SEM images of (**a**) LS, (**b**) CLS, and (**c**) CS.

**Figure 5:**
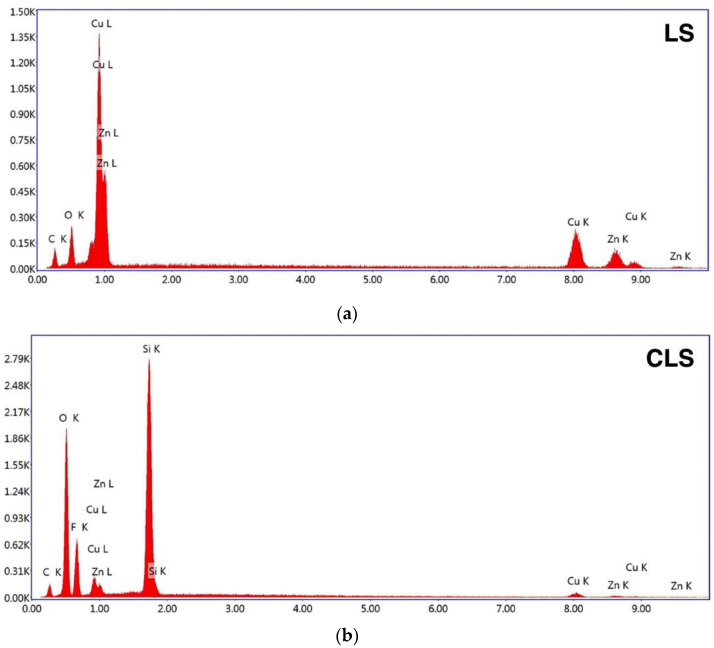
EDS spectrum of (**a**) LS and (**b**) CLS.

The contact angle measurements were performed on all samples as shown in Figure 2, and the results of the averaged apparent contact angles are shown in Table 1. The averaged apparent contact angles of the 10 µL water droplet on BS, LS, CS, and CLS were 90.9 ± 3.8°, 124.9 ± 8.9°, 153.5 ± 1.1°, and 164.5 ± 1.1°, respectively. Thus, BS and LS were hydrophobic, whereas CS and CLS, with apparent contact angles of greater than 150°, were superhydrophobic. In addition to the apparent contact angle, the low contact angle hysteresis of 8.66 ± 1.23° and 4.19 ± 2.43° were recorded for CS and CLS, respectively. In order to understand the surface structure differences among the samples, surface morphology was characterized with 3D microscopy and SEM. The 3D surface analysis was performed on the laser-treated samples. As shown in Figure 3, a groove pattern with a depth of 33 µm and width of 70 µm was successfully fabricated on the substrate. The laser ablation increases the roughness of the surface, leading to an increase in the apparent contact angle [38,39], allowing the droplet to rest on the groove structure, as observed in the SEM image shown in Figure 4a. LS and CLS samples were treated with nanolaser, resulting in identical groove patterns. In addition to BS and LS, sphere-like structures were observed on the CS and CLS samples, indicating that the silica nanoparticles were successfully coated, as shown in Figure 4b,c. The thickness of the spray-coated silica nanoparticle layer was 309.17 ± 20.82 nm. Although CS showed an apparent contact angle of 153.5 ± 1.1° due to the silica nanoparticle coating alone, CLS exhibited the greatest apparent contact angle of 164.5 ± 1.1°, as a result of the combination of laser processing and silica nanoparticle coating. The fabrication results were also confirmed with the EDS analysis as shown in Figure 5a,b. Cu, Zn, C, and O were present in the LS structure, whereas Si and F from the TEOS-FAS solution were detected in both CS and CLS. Based on the surface examination of the apparent contact angle, the SEM images, and EDS, it can be concluded that an increase in roughness due to laser ablation is responsible for the considerable rise in the apparent contact angle. The presence of silica nanoparticles on the surface of CS and CLS makes the samples highly water-repellent surfaces.

### 4.2. Anti-Icing Performance

**Figure 6:**
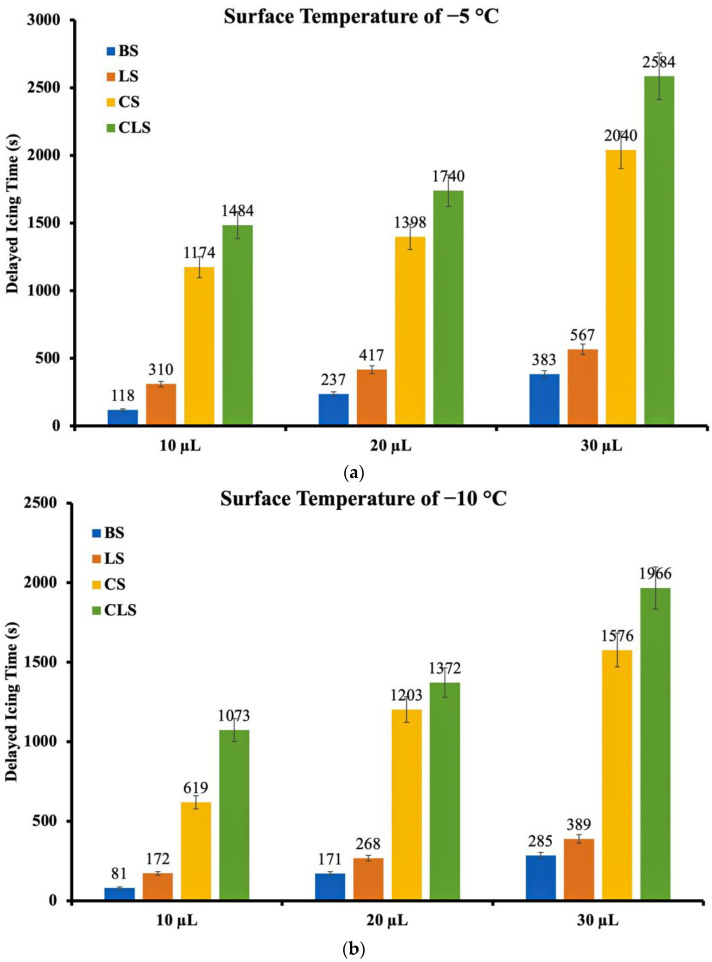

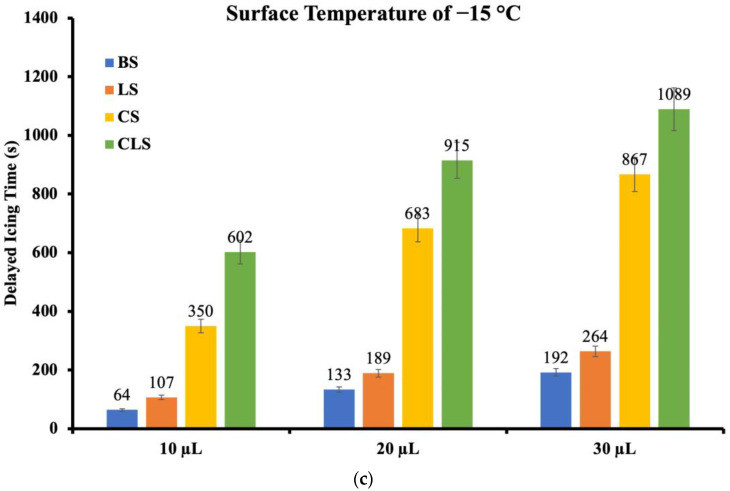
The delayed icing time of different droplet sizes on BS, LS, CS, and CLS at the surface temperatures of (**a**) −5 °C, (**b**) −10 °C, and (**c**) −15 °C.

The anti-icing experiment was performed on BS, LS, CS, and CLS at the temperatures of −5 °C, −10 °C, and −15 °C with droplet volumes of 10 µL, 20 µL, and 30 µL. As shown in Figure 6, the icing period increased with an increase in the droplet size at a given surface temperature, and decreased with a decrease in the surface temperature at a given droplet size. This was true for all experimented surfaces. Additionally, the results clearly demonstrated the effects of the surface structure on delaying the freezing time. When the apparent contact angle of the surface increased, the delayed icing time also increased. For example, at the surface temperature of −5 °C and the droplet size of 10 µL, the delayed icing time on CLS was 1484 s, whereas the delayed icing times of BS, LS, and CS were 118 s, 310 s, and 1174 s, respectively. It should be noted that the error bars in Figure 6 were associated with the cold plate power, the thermocouple device, the ambient temperature and humidity, and the droplet volume from the micropipettes. 

The findings also showed that when the droplet size increased, the increase in the delayed icing time was greater on CLS when compared to other surfaces. At the surface temperature of −5 °C, although the delayed icing time on BS from the 10 µL droplet size to the 30 µL droplet size increased by 265 s, the increase on CLS was 1100 s which was approximately 315% greater. To elaborate on the relationship between the increase in the droplet size and the increase in the delayed icing time, the experimental results of the surface temperature of −5 °C for the droplet sizes of 10 µL and 30 µL are given as an example. At the surface temperature of −5 °C and the droplet size of 10 µL, the delayed icing time on CLS was 1484 s or about 24 min which was longer than those on BS, LS, and CS by 1366 s (approximately 22 min), 1174 s (approximately 19 min), and 310 s (approximately 5 min), respectively. When the droplet size increased from 10 µL to 30 µL, the delayed icing time of CLS increased by 74% to 2584 s or about 43 min. It should be noted that this is the longest delayed icing time recorded in this study. Moreover, the 2584 s delayed icing time was longer than those on BS, LS, and CS at the same temperature and the same droplet size by 2201 s (approximately 36 min) or 575%, 2017 s (approximately 33 min) or 356%, and 547 s (approximately 9 min) or 27%, respectively. 

Apart from the droplet size, the surface temperature was also found to play a role in the delayed icing time. When the surface temperature decreased from −5 °C to −10 °C and −15 °C, the delayed icing time of all surfaces at all droplet sizes decreased. For the 30 µL droplet size, the delayed icing times on CLS at −10 °C and −15 °C decreased to 1966 (approximately 32 min) and 1089 s (approximately 18 min), accounting for 24% and 58% shorter than that at −5 °C, respectively. 

The study also showed that the surface structure played a more crucial role in achieving the anti-icing property when compared to the surface temperature or the droplet size. Although a lower surface temperature and a smaller droplet size accelerated the droplet freezing time, the effects of the surface structure were more significant. It can be observed that CLS at −15 °C still showed better anti-icing performance when compared with BS at a higher surface temperature and at a bigger droplet size. At the surface temperature of −5 °C, the delayed icing times of the droplet on BS at the droplet sizes of 10 µL and 30 µL were only 118 s and 383 s, respectively. In contrast, with the much lower surface temperature of −15 °C, at the droplet size of 10 µL, although the shortest delayed icing time on CLS reported in this study was 602 s, it was 410% longer than that on BS at −5 °C at the same droplet size, and 57% longer than that on BS at −5 °C at the droplet size of 30 µL. Moreover, the shortest delayed icing time on CLS at the lowest temperature and the smallest droplet size was longer than those on BS and LS at all conditions. 

The overall results in the current experiment are in line with those in the previous studies [6,7,8,9,10,11,12] even though different surface fabrication methods and substrate materials were utilized. Similar to [6,7,8,9,10,11,12], the results of this study also showed that the surface structure with a greater apparent contact angle resulted in a longer delayed icing time. 

**Figure 7:**
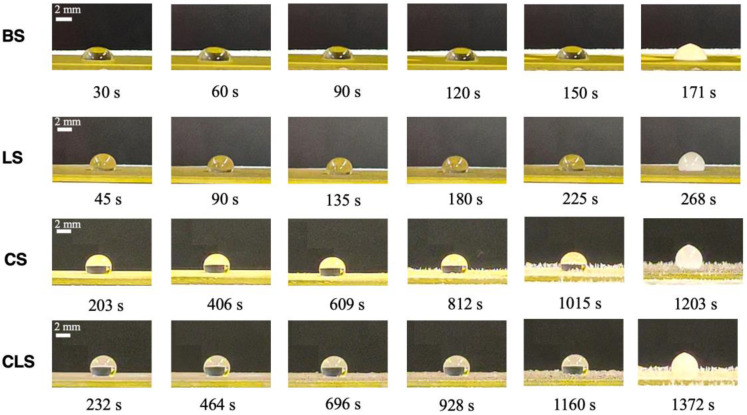
The delayed icing time at −10 °C with droplet size of 20 µL of BS, LS, CS, and CLS.

In order to elaborate on the effects of the laser processing and spray coating on the surface characteristics, the images of droplet shapes and the delayed icing times at −10 °C and the droplet size of 20 µL are shown in Figure 7. BS showed the shortest delayed icing time due to its lowest apparent contact angle. When a water droplet was placed on BS, the droplet spread across the surface and had a large contact area, causing it to quickly solidify into ice. LS showed a longer delayed icing time compared to BS due to the increased apparent contact angle from 90.9° to 124.9°. This was possible because of the laser-induced pattern on the sample, which increased surface roughness and decreased the contact area between the droplet and the solid substrate. In addition, CS demonstrated an even longer delayed icing time as a result of the presence of silica nanoparticles, which further decreased the solid-liquid contact area. CLS showed the most excellent anti-icing property due to its large apparent contact angle between the water droplet and the sample surface. This large apparent contact angle reduced the solid–liquid contact area, resulting in a decrease in the heat transfer between the droplet and the substrate. It led to a significant increase in the delayed icing time. 

Apart from the surface characteristics, the results of the study can be explained with heat transfer mechanisms. Heat transfer occurs on a droplet resting on a solid substrate in several ways. A droplet gains heat from the surrounding air via thermal radiation, and loses heat through (i) thermal conduction between the droplet and the substrate surface, and (ii) the cooled air trapped below the droplet due to thermal conduction and thermal convection [20,21]. It is assumed that the droplet maintains its spherical shape, the droplet size is constant, and the surrounding environment remains unchanged. The heat transfer equation describing the relationship between thermal gain and loss can be written as follows:

(1) $$\frac{dE_{dl}}{dt}={\dot{E}}_{r,air}-{\dot{E}}_{c,sub}-{\dot{E}}_{cv,cair}$$

The leftmost term represents the change in the heat transfer rate of the droplet. ${\dot{E}}_{r,air}$ is the heat transfer rate due to thermal radiation between the droplet and the surrounding environment, and ${\dot{E}}_{c,sub}$ and ${\dot{E}}_{cv,cair}$ are the thermal conduction heat transfer rate between the substrate and the droplet, and the thermal conduction and thermal convection caused by the cooled air trapped between the droplet and the cooled substrate, respectively. In the case of BS, with the apparent contact angle of 90.9 ± 3.8°, the contact area of the droplet on the substrate is large with no trapped air, and the contact area of the droplet exposed to the environment is small. Thus, ${\dot{E}}_{cv,cair}$ is approximately zero, and ${\dot{E}}_{r,air}$ is relatively very small. Meanwhile, ${\dot{E}}_{c,sub}$ is significantly high and dominates the cooling droplet. When the substrate is modified with silica nanoparticles, the apparent contact angle of the droplet on the substrate significantly increases, leading to an increase in the contact area between the droplet surface and the surrounding environment, a significant reduction in the contact area between the droplet surface and the substrate, and a presence of the cooled air between the cooling droplet and the cold substrate. As a result, for CS, ${\dot{E}}_{r,air}$ and ${\dot{E}}_{cv,cair}$ increase, whereas ${\dot{E}}_{c,sub}$ dramatically decreases. In addition, given that air has much smaller thermal conductivity than the solid substrate and its thermal convection is relatively small, although ${\dot{E}}_{cv,cair}$ appears for the case of CS, its amount is much smaller than the decrease in ${\dot{E}}_{c,sub}$. As a result, the change in the heat transfer rate of the droplet on CS is much smaller than that on BS, leading to a much longer delayed icing time. Moreover, when both the laser treatment and the spray coating are applied to the substrate, the apparent contact angle of the droplet on CLS increases and is larger than that on CS. Therefore, ${\dot{E}}_{c,sub}$ of CLS further decreases, resulting in extended delayed icing time. When the droplet size increases, the surface area between the droplet and the environment also increases, but the contact area between the droplet and the substrate approximately remains the same. Therefore, ${\dot{E}}_{r,air}$ increases and the droplet gains more heat through the thermal radiation, resulting in an increase in the delayed icing time.

## 5. Conclusions

A bare surface (BS), a lasered surface (LS), a coated surface (CS), and a coated-lasered surface (CLS) were fabricated and used in this study. All surfaces were successfully fabricated and were inspected and analyzed using contact angle measurement, 3D microscopy, scanning electron microscopy (SEM), and energy-dispersive spectroscopy (EDS). The anti-icing performances of the four different brass substrates were experimentally investigated with varying surface temperatures and droplet sizes. The findings showed that the deposition of silica nanoparticles on the nanolasered surface gave better superhydrophobicity when compared with the smooth surface deposited with silica nanoparticles or the rough surface created by nanolaser treatment. Although the apparent contact angles of BS, LS, and CS were 90.9 ± 3.8°, 124.9 ± 8.9°, and 153.5 ± 1.1°, respectively, CLS showed the best superhydrophobic property with the largest apparent contact angle of 164.5 ± 1.1°. Both CS and CLS also showed low contact angle hysteresis. The findings also revealed that as the apparent contact angle increased, the time taken for a liquid droplet to freeze was longer due to the reduced contact area between the droplet and the cold substrate. At the fixed surface temperature, an increase in the droplet size also resulted in an increase in the delayed icing time because the surface area increased, which led to more heat gain from the surrounding air. Conversely, with the same droplet size, the delayed icing time decreased as the surface temperature decreased. In addition, when the droplet size increased, the increase in the delayed icing time was more significant on CLS than on other surfaces. The CLS surface exhibited the longest delayed icing time under all conditions. For a 30 µL droplet size at −5 °C, the longest delayed icing time on CLS recorded in this study was 2584 s or about 43 min. It was 575%, 356%, and 27% longer than the delayed icing times on BS, LS, and CS, respectively. Moreover, the shortest delayed icing time on CLS at the lowest temperature and the smallest droplet size was longer than the delayed icing times on BS and LS at all conditions. Based on the results from the experiment, the shortest delayed icing time of CLS which occurred at the lowest surface temperature of −15 °C and at the smallest droplet size of 10 µL was 410% longer than the delayed icing time of BS at the highest surface temperature of −5 °C at the same droplet size. Moreover, when compared to BS with the biggest droplet size of 30 µL, the shortest delayed icing time of CLS was still 57% longer. This clearly shows that the combined fabrication method of nanolaser ablation and spray coating of silica nanoparticles plays a significant role in delaying the phase change of water droplets on subzero-temperature surfaces. Moreover, this implies that the effects of surface structure are more significant than the surface temperature and droplet size. In addition to the experimental work, the results can also be explained with the heat transfer model. With the very high apparent contact angle of CLS, thermal conduction between the solid–liquid contact area decreases, leading to much longer delayed icing time. Additionally, when the droplet size increases, the droplet gains more heat through thermal radiation, resulting in an increase in delayed icing time. 

## Figures and Tables

**Table 1:** Averaged apparent contact angles and fabrication methods of all samples.

Surfaces	Fabrication Methods	Averaged Apparent Contact Angle
BS	Polished	90.9 ± 3.8°
LS	Polished + Lasered	124.9 ± 8.9°
CS	Polished + Spray Coated	153.5 ± 1.1°
CLS	Polished + Lasered + Spray Coated	164.5 ± 1.1°

## Data Availability

The data presented in this study are available upon request from the corresponding author.
